# An assessment of tropical cyclones rainfall erosivity for Taiwan

**DOI:** 10.1038/s41598-019-52028-5

**Published:** 2019-11-01

**Authors:** Jayalakshmi Janapati, Balaji Kumar Seela, Pay-Liam Lin, Pao K. Wang, Utpal Kumar

**Affiliations:** 1 Institute of Atmospheric Physics, Department of Atmospheric Sciences, National Central University, Jhong-Li Region, Tao-Yuan City, Taiwan; 2 Taiwan International Graduate Program (TIGP), Earth System Science Program, Research Center for Environmental Changes, Academia Sinica, Taipei, Taiwan; 3 Earthquake-Disaster & Risk Evaluation and Management Center, National Central University, Jhong-Li Region, Tao-yuan City, Taiwan; 4 Department of Atmospheric and Oceanic Sciences, University of Wisconsin-Madison, Madison, Wisconsin USA; 5 Research Center for Environmental Changes, Academia Sinica, Taipei, Taiwan; 6 Taiwan International Graduate Program (TIGP), Earth System Science Program, Institute of Earth Sciences, Academia Sinica, Taipei, and National Central University, Jhong-Li Region, Tao-Yuan City, Taiwan

**Keywords:** Hydrology, Natural hazards

## Abstract

Rainfall erosivity (or water erosion) has severe implications on agriculture, water, and land use management. Though, there were Rainfall erosivity studies on regional and global scale, tropical cyclones’ Rainfall erosivity is poorly assessed and have not been documented for one of the most cyclones affecting regions of the world like Taiwan. Here, using 15-years of raindrop size distributions (RSD) and 60-years of hourly rain gauges data, we estimated cyclones (also called typhoons) rainfall erosivity over Taiwan, and establish that typhoons’ mean rainfall erosivity is higher than the global mean rainfall erosivity. Moreover, regional variability of typhoons rainfall erosivity showed an increasing pattern from north to south (Taipei to Pingtung), with relatively higher values over eastern and southern parts of Taiwan. The annual mean erosivity of typhoons rainfall showed raising trends over eastern and southern Taiwan during 1958–2017. Our results provide an insight in assessing the land use and agricultural management for Taiwan.

## Introduction

Tropical cyclones cause devastating loss of life and property globally^1,2^. Taiwan is one of the most intense tropical cyclones (typhoons) prone areas in the world^3^ with an average number of 3–4 typhoons striking the island every year^4^. Typhoons’ rainfall extremes over Taiwan are accountable for more sediment discharge^5,6^, natural hazards like floods, debris flow, and landslides^7–10^. Typhoons-induced rainfall kinetic energy (KE) is the driving force for surface runoff and landslide, and can be used as a proxy to determine the landslides triggering^11^. The rainfall KE is the key parameter in the estimation of rainfall erosivity factor (or R-factor), a substantial factor for soil erosion risk assessment in Universal Soil Loss Equation (USLE), Revised Universal Soil Loss Equation (RUSLE), and RUSLE2^12–15^. The ability of rainfall to cause soil erosion can be described in terms of R-factor, and its variability immensely influences the agriculture, water, and land use management. The R-factor of a given location is defined as the annual accumulation of EI_30_ index (in M J mm ha^−1^ h^−1^): the product of kinetic energy of each event (E) and its maximum 30-min rainfall intensity (I_30_)^12,14^. An apprehending of R-factor can prominently improves an accurate assessment of soil erosion.

Since the direct measurement of rainfall KE requires a precise and expensive experimental setup^16,17^, alternative approaches have been developed to compute the rainfall KE from rainfall intensity (I)^18,19^, which require empirical relations between KE and I. As the KE of raindrop is proportional to the third power of diameter and square of fall velocity, the combined information of raindrop size and fall velocity of rain allows us to derive KE-I relations^18–21^. The KE-I relations have been developed in the form of linear^22^, exponential^18,23^, power^24^, and logarithmic^25^, which are effective for the observational site or to the regions with similar geographical and meteorological characteristics^21,23–25^, and vary with geographical location, local climate and precipitation microphysics^18,20,23^.

Although, most of the previous studies on R-factor were devoted to seasonal or annual basis at regional, national, continental and global level^26–31^, limited studies were reported on the basis of storm types^32^, even such study has the limitation due to the adaptation of elsewhere KE-I relations in evaluating the R-factor. Here ‘elsewhere’ refers to the other country’s KE-I relations. Due to the paucity of local KE-I relations, vast majority of rainfall erosivity studies have adopted elsewhere KE-I relations^15,31,32^ and only limited researchers used their local KE-I relations^33^. Similar scenario can be seen even for Taiwan in estimating the typhoons rainfall KE/erosivity^11^, which may lead to overestimation or underestimation of typhoons rainfall erosivity for this island^19,33–35^. Though, there were reports on local KE-I relations for Taiwan, they are limited to the seasonal rainfall or combination of monsoon and typhoon rainfall^36,37^. Moreover, the raindrop size distribution (RSD) characteristics of tropical cyclones and their KE-I relations were found to be different from that of the non-tropical cyclones precipitation^19,38–40^. Henceforth, it is crucial to explore Taiwan typhoons R-factor by adopting indigenous KE-I relations.

Here, we use the long-term RSD of 65 typhoons (2002–2017) and dense network of hourly rain gauges data (1958–2017). We investigate and evaluate the KE-I relations for typhoon rainfall, and demonstrate that the power form of KE-I relation is appropriate. Furthermore, the rainfall erosivity (R-factor) and erosivity density (R-factor density) are assessed for 393 typhoon rainfall events (occurred during 1958–2017) by adopting the estimated power form of KE-I relation to all rain gauge stations distributed over Taiwan. Also, trends in typhoons’ rainfall erosivity across the Island are discussed.

## Results

The rainfall statistics of 75 typhoon rainfall erosive events computed from RSD measurements of disdrometer during 2002–2017 are summarized in Table 1. The rainfall depth, number of particles, duration of the event, maximum and mean rainfall intensity of these 75 typhoon events varies from 10.43–355.75 mm, 31175–1615753, 159–6900 min, 9.97–143.17 mm h^−1^ and 0.24–14.22 mm h^−1^, respectively. The number of particles range, 31175 to 1615753, represents the number of raindrops recorded by the JWD for 75 typhoon rainfall events. Out of 75 typhoon rainfall events, we used 72 events (70600 data points) to establish the empirical relations between kinetic energy (Kinetic energy expenditure - KE_time,_ and kinetic energy content - KE_mm_) and rainfall intensity (I) by using nonlinear least square regression analysis. The rest three events (Fungwong, Lupit, and Namtheun: designated with * symbol, in bold and italic font in Table 1) are used to validate the derived KE-I (KE_time_-I and KE_mm_-I) relations.

**Table 1:** Rainfall statistics (Date, duration, no. of particles, rainfall depth, and intensity) of 75 typhoon rainfall erosive events observed by disdrometer.

S. No.	Typhoon name	Date and start time	Duration (minutes)	No. of particles	Rainfall depth (mm)	Rainfall intensity (mm h^−1^)		
						Maximum	Mean	Standard deviation
1	Sinlaku	5/9/2002 15:06	1907	285175	32.28	38.35	1.02	2.51
2	Mindule	2/7/2004 1:00	1649	586270	90.71	56.47	3.3	6.23
3	Rananim	10/8/2004 19:15	1614	323349	77.17	86.8	2.87	10.12
4	Aere	23/08/04 09:35	2631	656241	228.97	76.36	5.22	8.12
5	Songda	5/9/2004 6:10	230	52118	10.43	46.03	2.72	6.13
6	Songda	6/9/2004 0:09	159	54525	18.89	57.36	7.13	11.94
7	Haima	8/9/2004 1:10	661	153702	12.16	34.72	1.1	3.04
8	Haima	9/9/2004 14:10	487	98361	37.68	70.06	4.64	10.28
9	Haima	10/9/2004 12:10	3205	1564637	277.54	98.06	5.2	10.87
10	Meari	25/09/04 05:40	1542	183809	13.86	26.54	0.54	1.98
11	Tokge	18/10/04 10:12	1702	835644	21.47	9.97	0.76	1.13
12	Nock-ten	24/10/04 01:21	3611	927626	84.8	96.23	1.41	5.07
13	Nanmdol	3/12/2004 1:53	2257	543890	67.57	33.83	1.8	2.7
14	Haitang	17/07/05 06:59	3687	925339	222.61	88.86	3.62	8.25
15	Matsa	4/8/2005 3:51	2437	750259	217.67	101.06	5.36	9.75
16	Talim	31/08/05 01:40	1804	280064	84.87	67.72	2.82	6.84
17	Khanun	10/9/2005 11:10	854	213791	12.89	30.06	0.91	2.54
18	Darmey	22/09/05 01:04	1502	221685	45.98	55.97	1.84	4.61
19	Longwong	1/10/2005 8:52	1477	261609	60.66	101.94	2.46	8.17
20	Chanchu	16/05/06 02:27	2570	236904	32.29	12.31	0.75	1.88
21	Chanchu	19/05/06 00:38	482	59018	11.94	35.87	1.49	4.09
22	Ewiniar	8/7/2006 20:06	759	175041	65.09	118.67	5.15	14.64
23	Bilis	12/7/2006 9:15	5053	732898	70.73	54.55	0.84	2.71
24	Kaemi	25/07/06 14:24	1660	77646	18.02	52.37	0.65	3.42
25	Saoma	8/8/2006 12:34	1115	93451	15.34	49.68	0.83	3.83
26	Bopha	9/8/2006 23:57	726	132751	24.11	61.19	1.99	6.55
27	Shanshan	12/9/2006 01:16	1364	191698	54.3	92.04	2.39	7.01
28	Shanshan	13/09/06 06:37	500	131165	32.39	68.11	3.89	9
29	Shanshan	15/09/06 07:17	3644	435828	39.95	65.78	0.66	2.73
30	Pubak	7/8/2007 09:00	2220	77064	10.83	11.59	0.29	0.82
31	Wutip	14/08/07 08:30	382	81251	43.29	63.56	6.8	16.37
32	Sepat	17/08/07 13:07	3209	129099	19.04	24.65	0.36	1.23
33	Wipha	17/09/07 06:01	3733	968851	96.16	49.19	1.55	3.3
34	Mitag	26/11/07 03:40	2660	574797	74.29	22.47	1.68	2.41
***35***	* Fungwong	***26/07/08 22:26***	***4015***	***335743***	***124.67***	***143.17***	***1.86***	***10.14***
36	Sinlaku	11/9/2008 06:02	6676	1615753	355.75	83.96	3.2	7.55
37	Jangmi	27/09/08 02:10	4552	586699	140.82	86.5	1.86	4.61
38	Parma	4/10/2009 10:30	3690	495127	65.35	16.89	1.06	1.83
***39***	* Lupit	***22/10/09 10:29***	***4361***	***678629***	***27.7***	***12.95***	***0.38***	***0.99***
***40***	* Namtheun	***30/08/10 02:28***	***247***	***52710***	***26.59***	***122.77***	***6.46***	***21.26***
41	Namtheun	30/08/10 13:30	315	215043	74.65	92.85	14.22	18.51
42	Meranti	10/9/2010 16:13	359	57103	26.14	70.17	4.37	11.77
43	Megi	18/10/10 02:40	6900	973252	102.58	37.1	0.89	2.12
44	Songda	27/05/11 09:38	1914	604537	19.04	21.5	0.6	1.33
45	Sarika	10/6/2011 16:32	304	31175	15.03	75.47	2.97	10.04
46	Meari	24/06/11 17:37	1784	205498	26.36	14.65	0.89	2.02
47	Muifa	6/8/2011 17:39	639	68809	22.09	54.83	2.07	7.52
48	Talim	18/06/12 13:21	4711	789295	85.89	65.97	1.09	3.4
49	Saola	30/07/12 00:25	5500	1199496	296.2	81.02	3.23	7.33
50	Haikui	6/8/2012 7:03	458	62019	11.55	45.55	1.51	4.61
51	Tembin	22/08/12 08:45	2181	304661	105.94	97.66	2.91	11.06
52	Bolavin	26/08/12 00:50	1390	93594	22.39	62.61	0.97	4.68
53	Sanba	14/09/12 00:25	2855	315352	130.45	93.31	2.74	9.22
54	Bopha	8/12/2012 0:00	1960	622973	40.19	13.04	1.23	1.52
55	Soulik	12/7/2013 0:48	2037	326205	85.41	42.12	2.52	4.87
56	Soulik	14/07/13 07:25	1821	79771	12.82	22.26	0.42	1.6
57	Kongrey	28/08/13 00:24	2856	545091	111.84	104.98	2.35	8.02
58	Toraji	30/08/13 00:00	3469	719941	46.65	54.87	0.81	2.47
59	Usagi	19/09/13 19:16	4002	270513	44.07	58.44	0.66	3.69
60	Fitow	4/10/2013 0:29	3691	300156	31.2	68.99	0.51	2.93
61	Matmo	22/07/14 08:00	2400	244233	55.24	48.12	1.38	3.03
62	Fungwang	21/09/14 08:00	1080	194242	89.98	123.4	5	13.79
63	Vongfong	8/10/2014 17:39	4660	422190	39.18	54.11	0.5	2.48
64	Noul	11/5/2015 0:18	1422	85864	16.45	56.4	0.69	4.24
65	Chanchom	9/7/2015 2:05	2225	337854	42.67	63.18	1.15	4.41
66	Soudelor	6/8/2015 11:29	322	37851	19.87	91.08	3.7	13.05
67	Soudelor	7/8/2015 0:00	1301	213187	73.9	69.41	3.41	7.02
68	Goni	22/08/15 13:12	2088	104937	17.69	86.26	0.51	4.14
69	Goni	24/08/15 20:58	344	165002	22.11	47.36	3.86	6.14
70	Dujuan	26/09/15 03:52	4088	976602	91.77	52.2	1.35	3.6
71	Meranti	12/9/2016 1:52	3263	326550	13.32	35.43	0.24	1.17
72	Malakas	16/09/16 07:18	2442	592981	45.43	52.86	1.12	3.54
73	Megi	26/09/16 02:46	2595	589245	136.59	66.95	3.16	6.62
74	Nesat/Haitang	29/07/17 01:47	1333	113150	13.59	22.35	0.61	1.44
75	Talim	12/09/17 16:47	2113	196436	21.93	69.06	0.62	3.59

The “No. of particles” column represents the number of raindrops measured by the disdrometer for each typhoon event.

### Establishment of kinetic energy-rainfall intensity (KE-I) relations

**Figure 1:**
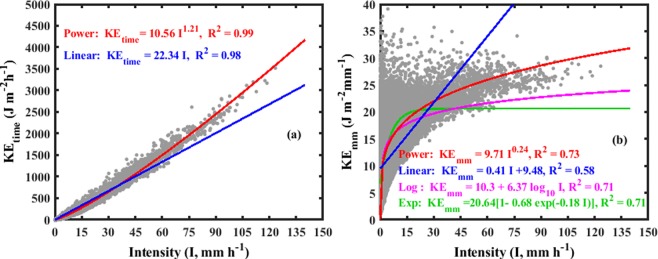
Scatter plot of typhoon rainfall (**a**) kinetic energy expenditure (KE_time_) and Intensity (I) fitted with linear and power laws (**b**) kinetic energy content (KE_mm_) and Intensity (I) fitted with linear, power, logarithmic and exponential equations for 73 typhoon rainfall events (70600 1-min data points).

Figure 1a depicts the scatter plot for kinetic energy expenditure (KE_time_) and intensity (I) data points (70600) for 72 typhoon rainfall events. The KE_time_ ranges from 0.1–3517.9 J m^−2^ h^−1^, corresponding to rainfall intensities of 0.1–123.4 mm h^−1^. The mean value of KE_time_ is 72.65 J m^−2^ h^−1^ with standard deviation of 194.75 J m^−2^ h^−1^. The best estimates for power^24,41^ and linear^22^ forms of KE_time_-I relations from the regression analysis are given Fig. 1a. The regression line derived for the data points showed relatively higher coefficient of determination for both power and linear equations. Even so, for rainfall intensity >50 mm h^−1^, the KE_time_ is underestimated by the linear equation and the power equation created good estimates with its regression line approximately passing through middle of data points, and the data points are relatively least scattered with uniform distribution over power regression line.

A total number of 70600 one-min data points of kinetic energy content (KE_mm_) and intensity (I) are shown in Fig. 1b. The KE_mm_ varies from 1.05–39.11 J m^−2^ mm^−1^ for the rainfall intensity values of 0.1–123.4 mm h^−1^ with mean and standard deviation of 11.14 J m^−2^ mm^−1^ and 5.79 J m^−2^ mm^−1^, respectively. The maximum KE_mm_ of current typhoons is higher than the global average maximum KE_mm_ (28.3 ± 2.9 J m^−2^ mm^−1^)^20^. The KE_mm_ data points are more scattered at lower rainfall intensities (<15 mm h^−1^) and are getting narrowed with the increase of rainfall intensity. By excluding the higher KE_mm_ values at lower rainfall intensities (<15 mm h^−1^), a maximum KE_mm_ of 35.71 J m^−2^ mm^−1^ is noticed. For rainfall intensity greater than 75 mm h^−1^, the KE_mm_ ranges from 23.61–34.11 J m^−2^ mm^−1^ with mean and standard deviation of 26.4 J m^−2^ mm^−1^, 1.61 J m^−2^ mm^−1^, respectively. The established power, linear, logarithmic^25^, and exponential^18,23^ fitting curves for KE_mm_ and I are depicted in Fig. 1b.

Among four models (Fig. 1b), power model showed relatively higher performance, and other two models (exponential and logarithmic) are significantly underestimated the KE_mm_ for rainfall intensities >30 mm h^−1^. The exponential form of KE_mm_-I relation in the present study is different from that of the Chang *et al*.^37^ (KE_mm_ = 32.19 [1-0.725e^−0.029I^]), which is due to their collective consideration of both seasonal and typhoon rainfall RSD in deriving KE_mm_-I relations. Present exponential form of KE_mm_-I relation is also found to be different from that of the Van Dijk *et al*.^20^ generalized equation (KE_mm_ = 28.3 [1-0.52 exp(−0.042I)]) and evidently shows that the KE-I relations of typhoon rainfall are different from that of the non-typhoon rainfall, and confirms that, there is need to adopt local tropical cyclones KE-I relations in computing the rainfall KE/erosivity of tropical cyclones of a given region.

The goodness-of-fit statistics in terms of coefficient of determination (R^2^), root mean square error (RMSE), and normalized RMSE (NRMSE) for power, linear, logarithmic and exponential equations of KE-I relations for 72 events are provided in Table 2. From the Table 2, amid four regression models (power, linear, logarithmic and exponential), statistically the power law showed higher predictive capability for the two erosivity indices (KE_time_ and KE_mm_, Fig. 1). Similar characteristics were reported by the previous researchers elsewhere^40^.

**Table 2:** Statistical parameters [correlation coefficient: R^2^, Root mean square error (RMSE), normalized RMSE] of 72 events and the three events selected for validation.

Typhoon	Statistical parameters	KE_time_-I		KE_mm_-I			
		Power	Linear	logarithmic	Exponential	Power	Linear
72 rainfall events	R^2^	0.99	0.98	0.71	0.72	0.72	0.58
	Rmse	23.10	40.28	4.07	4.05	3.99	4.72
	Nrmse	0.19	0.33	4.07	4.05	0.03	4.72
Fungwong	R^2^	0.99	0.99	0.72	0.71	0.76	0.65
	RMSE	65.15	135.87	4.68	4.72	4.36	5.74
	NRMSE	0.45	0.95	0.03	0.03	0.03	0.04
Lupit	R^2^	0.97	0.95	0.47	0.47	0.48	0.45
	RMSE	9.66	24.89	4.80	4.49	4.60	5.23
	NRMSE	0.75	1.94	0.37	0.35	0.36	0.41
Namtheun	R^2^	0.99	0.99	0.96	0.95	0.97	0.89
	RMSE	81.05	153.95	2.93	3.59	2.99	9.73
	NRMSE	0.66	1.25	0.02	0.03	0.02	0.08

### Validation of kinetic energy-rainfall intensity (KE-I) relations

**Figure 2:**
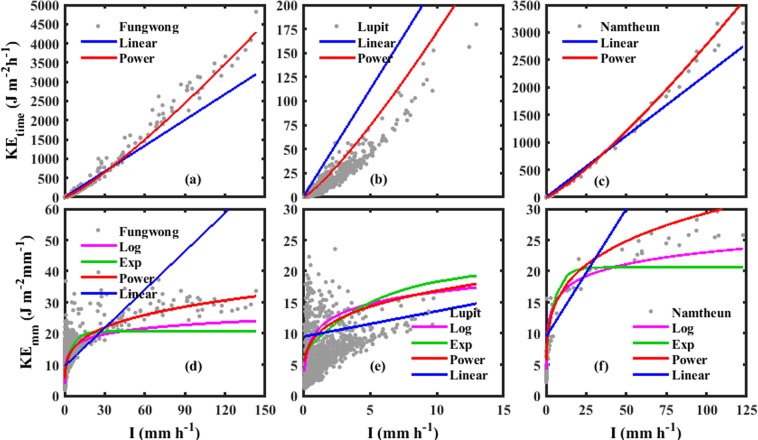
Validation of estimated KE_time_-I relations (**a**–**c**), KE_mm_-I (**d–f**) in the form of linear (blue color), power (red color), exponential (green color), and logarithmic (pink color) laws with Fungwong (Event no. 35), Lupit (Event no. 39), and Namtheun (Event no. 40) typhoon events, respectively.

It is important to endorse the estimated KE-I relations before using them in rainfall erosivity assessment, and so, we validated the derived empirical (KE-I) relations with three typhoon events (Event no. 35, 39, and 40 in Table 1). Among these three events, two events have higher rainfall intensities with long (Event no. 35. Fungwong) and short (Event no. 40. Namtheun) duration, and the third one (Event no. 39. Lupit) has long duration and relatively low rainfall intensities, but this is quite erosive if the whole event is considered. Figure 2a–c represents KE_time_ versus I for three events with linear and power relations. For Fungwong and Namtheun events (Fig. 2a,c), estimation of KE_time_ by power law showed a good performance and underestimated by linear law at higher rainfall intensity (>35 mm h^−1^). In the case of the Lupit event (Fig. 2b), even if both power and linear laws overestimated, the power law shows more predictive capability than liner law. The goodness-of-fit statistics (R^2^, RMSE, and NRMSE) for power and liner forms of KE_time_-I relations are quantified in Table 2. Even though two equations showed relatively higher coefficient of determination (R^2^), the power law showed lower RMSE and NRMSE, indicating that the power law has better performance than linear in estimating the KE_time_ from rainfall intensity.

Validation of power, linear, logarithmic, and exponential forms of KE_mm_-I relations with three events (Event no. 35, 39, and 40) are shown in Fig. 2d–f. In all the three events (Fig. 2d–f), power, logarithmic and exponential relations showed similar prediction capability for lower rainfall intensities (<5 mm h^−1^). Exponential and logarithmic fits severely underestimated the KE_mm_ for event no. 35 (for I > 30 mm h^−1^) and event no. 40 (for I > 40 mm h^−1^). The KE_mm_ is well predicted by the power equation for long duration- high intensity rainfall event (Event no. 35. Fungwong, Fig. 2d), nonetheless, KE_mm_ is overestimated for remaining two events (Event no. 39 and 40). The goodness-of-fit statistics (R^2^, RMSE, and NRMSE) for power, linear, logarithmic and exponential equations of KE_mm_-I relations are provided in Table 2 for the three typhoon events. Further, estimated KE-I models (KE_time_-I: linear, power, KE_mm_-I: linear, power, logarithmic, and exponential) are cross-validated by using leave p out cross validation method (where p = 3 events), and the iterations were performed for 1000 times. We found that the power model is more reliable than the other models for both KE_time_-I and KE_mm_-I relations.

Because of spurious self-correlations between KE_mm_ and I, and relatively higher KE_mm_ values at lower rainfall intensities, the KE_mm_-I relations lead to uncertainty in predicting the rainfall KE^21^ for larger I values. This characteristics is clearly persistent even for present typhoon rainfall events (Fig. 1b). Hence, we prefer to adopt KE_time_-I relation rather than KE_mm_-I relation in computing the rainfall erosivity and was strongly suggested^21^.

### Spatial variation of typhoon rainfall, rainfall erosivity and erosivity density

During 1958–2017, with Central Weather Bureau (CWB) typhoon warning periods, a total number of 423 typhoons were recorded by dense network of 711 rain gauge stations over Taiwan, and among 423 typhoons 393 were qualified for the erosive event criteria. Nevertheless, in the current study, in order to estimate the representative typhoons rainfall erosivity, rain gauge stations (288) with minimum record period of 20 years^14^, whose elevation ranges from 2–3844.8 km asl are adopted. These 288 rain gauge stations have typhoon events for a total number of 7227 years with a mean value of 25 years per station, with number of events ranging from 58 to 368. The erosivity factor (EI_30_) of 393 typhoons over 288 rain gauge stations are computed by applying the estimated KE-I relation.

**Figure 3:**
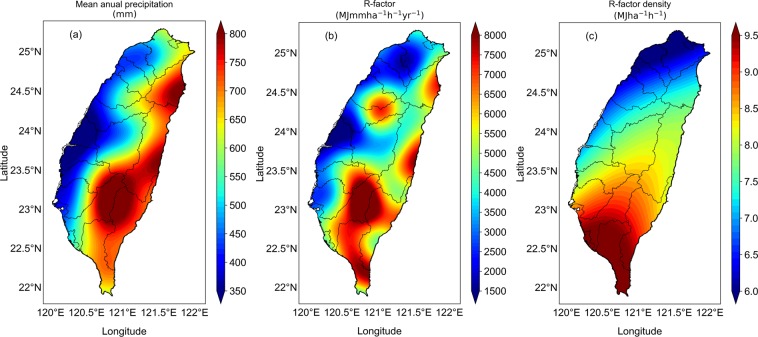
Spatial distribution of typhoons-induced (**a**) mean annual precipitation (**b**) R-factor, and (**c**) R-factor density map plotted with kriging over Taiwan during 1958–2017.

The annual mean precipitation, rainfall erosivity, and erosivity density of 393 typhoons over Taiwan are depicted in Fig. 3. The estimated stations’ annual mean precipitation ranges from 196.76 to 1427.3 mm yr^−1^, with mean and standard deviation of 578.97 mm yr^−1^ and 231.04 mm yr^−1^, respectively. The Island-wide gridded annual mean precipitation values are derived by applying kriging interpolation to stations’ annual mean precipitation. The gridded annual mean typhoons-induced rainfall varies from 288.57–877.79 mm yr^−1^, with mean and standard deviation value of 586.90 mm yr^−1^ and 155.44 mm yr^−1^, respectively (Fig. 3a). The spatial distribution of typhoons precipitation depicts relatively higher values over eastern and south eastern part of Taiwan. Different (north, east, central, and south) regions of Taiwan are depicted with different color in Supplementary Fig. 1b. The typhoons-induced event rainfall amounts contributed to a percentage of 14.3%, 25.7%, 29.1%, and 30.9%, respectively, for north, central, east, and south regions of Taiwan. The deep central mountain ranges (CMR) of Taiwan helps in enhancing the convective activity of typhoons while they cross it, resulting in relatively higher precipitation over eastern and south eastern part of Taiwan than other areas^42^. The strong easterly winds of typhoons are blocked by the CMR leading to less precipitation across west coast of Taiwan^43^. Annual mean precipitations of Taiwan Counties are detailed in Table 3.

**Table 3:** County wise mean and standard deviation values of accumulated precipitation, annual mean precipitation, EI_30_, R-factor and R-factor density.

County	No. of rain gauges	Number of years		Accumulated precipitation (mm)		Annual mean precipitation (mm yr^−1^)		EI_30_ (MJ ha^−1^ mm h^−1^)		R-factor (MJ ha^−1^ mm h^−1^ yr^−1^)		R-factor density (MJ ha^−1^ h^−1^)	
		Min.	Max	Mean	SD	Mean	SD	Mean	SD	Mean	SD	Mean	SD
Taipei	28	20	60	17269.3	15165.9	553.2	223.0	99874.0	108511.8	3095.5	1725.2	5.3	1.01
Tauyoun	8	20	26	10317.7	5363.0	453.5	248.9	56569.6	56277.3	2539.3	2752.2	4.7	1.95
Hsinchu	11	21	26	11432.2	5382.3	456.7	196.1	68901.7	62573.2	2720.5	2366.3	5.2	1.83
Yilan	23	20	59	18828.8	6299.0	781.8	228.9	147759.0	67813.5	6222.1	3023.5	7.8	2.66
Hualien	25	20	60	17682.9	6122.5	760.7	167.5	143527.1	74744.8	6184.0	2968.1	7.8	1.99
Taitung	17	20	60	19368.4	11110.3	703.2	170.8	145901.5	85452.8	5412.3	2336.4	7.4	1.91
Tainan	24	20	39	10604.4	3305.6	458.0	118.6	88351.5	48149.4	3804.4	1873.1	8.0	1.55
Kaohsiung	32	20	58	16089.5	6228.7	675.1	227.2	169071.1	92288.1	7127.7	3515.3	10.5	2.68
Pingtung	28	20	59	16500.1	6801.8	697.1	189.6	175038.0	97840.0	7441.0	3642.6	10.3	2.48
Miaoli	13	20	27	11554.1	4309.7	474.3	153.2	99973.8	56532.2	4088.8	2162.1	8.2	2.37
Taichung	13	20	57	11290.5	4613.7	426.4	195.1	101241.0	73276.8	4052.5	3824.4	8.6	3.14
Changhua	6	20	24	5517.5	582.9	254.2	14.5	31201.6	5918.3	1433.6	205.3	5.6	0.55
Nantou	35	20	60	12990.1	6947.6	499.5	137.8	98448.1	52217.4	3901.6	1981.5	7.7	2.76
Yunlin	12	20	24	7070.5	3143.2	323.4	130.3	49891.5	37044.1	2261.2	1565.6	6.6	1.55
Chiyayi	16	21	60	15732.1	13295.2	543.6	262.5	162113.1	174868.6	5481.0	4492.6	8.8	2.99

Where Min. Max, SD represents the minimum, maximum, and standard deviation, respectively.

The R-factor values of 288 rain gauges with record period of 20–60 years varied from 886.75–22653.03 MJ mm ha^−1^ h^−1^ yr^−1^, with mean and standard deviation value of, respectively, 4880.74 MJ mm ha^−1^ h^−1^ yr^−1^ and 3261.97 MJ mm ha^−1^ h^−1^ yr^−1^. The Island-wide gridded annual mean R-factor values are derived by applying kriging interpolation to stations’ annual mean R-factors. The gridded R-factor values differs from 1005.73–9787.41 MJ mm ha^−1^ h^−1^ yr^−1^, with mean and standard deviation value of 4905.83 MJ mm ha^−1^ h^−1^ yr^−1^ and 1882.69 MJ mm ha^−1^ h^−1^ yr^−1^, respectively (Fig. 3b). Regional variability of typhoons R-factor showed an increasing pattern from north to south, with relatively higher values over eastern and southern part of Taiwan (Fig. 3b). As mentioned in the above paragraph, typhoons-induced event rainfall amounts contribute to a percentage of 14.3%, 25.7%, 29.1%, and 30.9%, respectively, for north, central, east, and south regions of Taiwan. Similarly, typhoon-induced rainfall erosivity contributed to a percentage of 9.3%, 25%, 28.1% and 37.2%, respectively, for north, central, east, and south regions of Taiwan. Typhoons invading region of Taiwan is surrounded by two major typhoon paths over the northwest Pacific region. One path moves south of Taiwan in westward to the south China sea, and the other turns in the north direction toward either Japan or Korea, which pass through the east side of Taiwan^44^. Because of steeper height of the central mountain range (CMR) of Taiwan, which is extended from north to south of the Taiwan Island, majority of the typhoons are blocked by these CMR, resulting in higher rainfall amounts over eastern and southern part of Taiwan (Fig. 3a), subsequently, relatively higher erosivity values over eastern and southern part of Taiwan. The regions with higher R-factor over Taiwan are in order with the higher precipitations areas (Fig. 3a). In addition to that, a good correlation is observed between precipitation (P) and EI_30_ values at event as well as annual wise (Supplementary Fig. 4), and the EI_30_-P relations at event and annual level are estimated as, respectively, EI_30_ = 0.68 P^1.44^ (R^2^ = 0.80) and EI_30_ = 1.69 P^1.23^ (R^2^ = 0.86). For the mostly associated tropical cyclones rainfall over Pacific coast of Mexico, García-Oliva *et al*.^45^ perceived mean annual erosivity of 6525.2 MJ mm ha^−1^ h^−1^_._ Recently, Laceby *et al*.^32^ demonstrated that tropical cyclones contributed 22% of precipitation and 40% of rainfall erosivity over Fukushima region of Japan, and they illustrated that the annual mean precipitation and R-factor ranged from, respectively, 68 to 639 mm yr^−1^ (mean: 422 mm yr^−1^) and 142 to 4547 MJ mm ha^−1^ h^−1^ yr^−1^ (mean: 1462 MJ mm ha^−1^ h^−1^ yr^−1^). Storm wise variation of mean precipitation and EI_30_ ranged from 41–240 mm and 118–1695 MJ mm ha^−1^ h^−1^, respectively, (Table S1. of Laceby *et al*.^32^). However, for Taiwan region, event mean precipitation and EI_30_ of typhoons varied, respectively, from 12.5–3059.5 mm and 0.47–92144.9 MJ mm ha^−1^ h^−1^, which are greater than the values over Japan. This demonstrates that the typhoons rainfall over Taiwan has much influence with intense rainfall and higher EI_30_/R factor values. Further, annual mean precipitation, rainfall erosivity for each county are computed and given in Table 3. Counties’ mean accumulated precipitation varied from 5517.5–19368.39 mm yr^−1^ with minimum precipitation over Changhua and maximum over Taitung. Annual mean R-factor ranges from 1433.59–7441.02 MJ mm ha^−1^ h^−1^ yr^−1^ with minimum value over Changhua and maximum value over Pingtung. Lee and Lin^46^ estimated rainfall erosivity of 16560 storm events for Kaohsiung City and Pingtung County in south Taiwan. They found mean annual total rainfall between 1376–4070 mm yr^−1^ and annual rainfall erosivity between 15000–70000 MJ mm ha^−1^ h^−1^ yr^−1^. The discrepancy in annual mean rainfall and rainfall erosivity values between present study and Lee and Lin^46^ is might be due to their adaptation of elsewhere KE-I relations or due to different sampling (10-min) of the rainfall data.

In order to identify the regions with high risk due to erosive events, typhoons rainfall erosivity density (ratio of annual R-factor to the annual mean precipitation) is computed for each station. For all the 288 rain gauges stations, erosivity density varies from 3.11–19.17 MJ ha^−1^ h^−1^ with mean and standard deviation of 7.92 MJ ha^−1^ h^−1^, and 2.79 MJ ha^−1^ h^−1^, respectively. The Island-wide gridded R-factor density values are derived by applying kriging interpolation to stations’ R-factor density values. The R-factor density map of Taiwan (Fig. 3c) ranges from 4.57–9.68 MJ ha^−1^ h^−1^ with mean and standard deviation of 7.77 MJ ha^−1^ h^−1^ and 0.99 MJ ha^−1^ h^−1^, respectively. Areas with higher erosivity density are an indication of locations with higher rainfall intensity events with shorter duration^29^. Higher erosivity density values are noticed over southern part of Taiwan, which imply that southern part is most prone to the soil loss and high flood risk than other regions of Taiwan. The R-factor density values of each county of Taiwan are illustrated in Table 3. County wise R-factor density show minimum for Hsinchu (5.25 MJ ha^−1^ h^−1^) and maximum for Kaohsiung (10.52 MJ ha^−1^ h^−1^).

### Trends in typhoons precipitation and erosivity

Quite diverse results were reported for rainfall erosivity trends over different parts of the world on seasonal and annual scale^32,33,47–52^. For instance, Webster *et al*.^53^, Emanuel^1^, and Mei and Xie^54^ noticed an increasing trend in the intensity of tropical cyclones, and in contrast, other researchers^55,56^ claim small or no trends. For Taiwan region, Tu *et al*.^57^ demonstrated that an abrupt shift in typhoon count series from 3.3 (1970–1999) typhoons per year to 5.7 (2000–2006). Tu and Chou^58^ analyzed the frequency, intensity, and duration of typhoon-induced rainfall over Taiwan by considering 21 rain gauge stations distributed over this Island for July to October months during 1970–2010. They perceived a significant increase in typhoon-induced rainfall over Taiwan which is due to the increase in number of typhoon days that affecting the Island rather than number of typhoons that pass through the typhoons invading region (18N–29.5N, 116E–126E). Recently, trend analysis for typhoon-induced rainfall at six rain gauges stations (along west coast: Taipei, Taichung, and Tainan; along east coast: Hualien, Taitung, and Hengchun) located over Taiwan was carried out by Liang *et al*.^59^. They showed that variations in the typhoon-induced rainfall trends at the selected six stations are related to the poleward shift of tropical cyclones over northwest Pacific, which is due to the weakening of the steering flow and western north Pacific subtropical high. Albeit, long-term trends in global tropical cyclones properties (number, intensity, duration, and destructive potential) have been documented^1,53,54,60,61^, trends in erosivity triggered by tropical cyclones rests unidentified.

**Figure 4:**
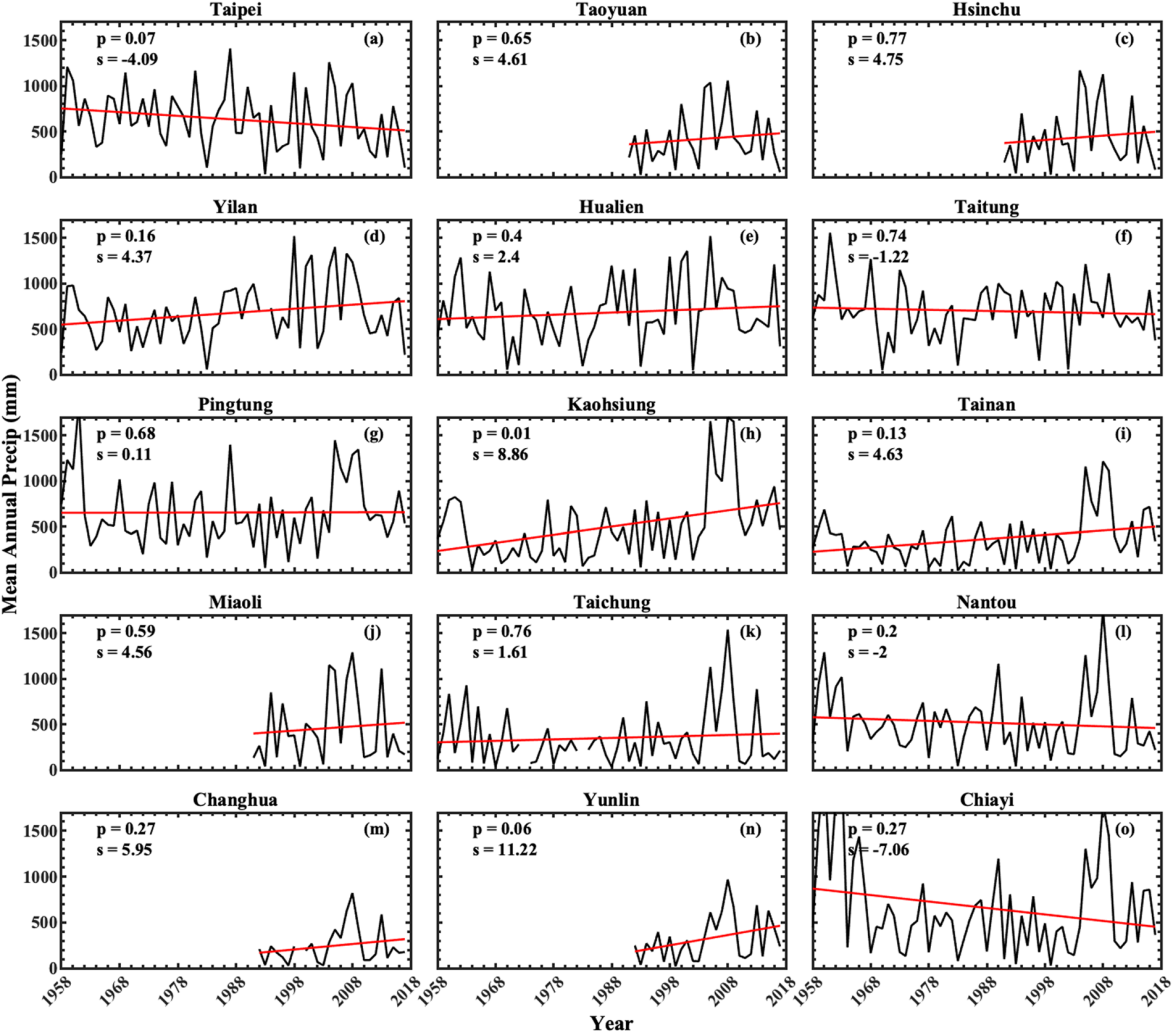
Time series of mean annual precipitation induced by typhoon rainfall events for 15 counties of Taiwan during 1958–2017. The rain gauge stations at Taoyuan, Hsinchu, and Maiaoli counties are available from the year 1991, and for Changhua and Yunlin from 1992. The slope of the trend line is depicted with ‘s’ in the legend. The gaps in mean annual precipitations lines denotes unavailability of data for the corresponding year.

**Figure 5:**
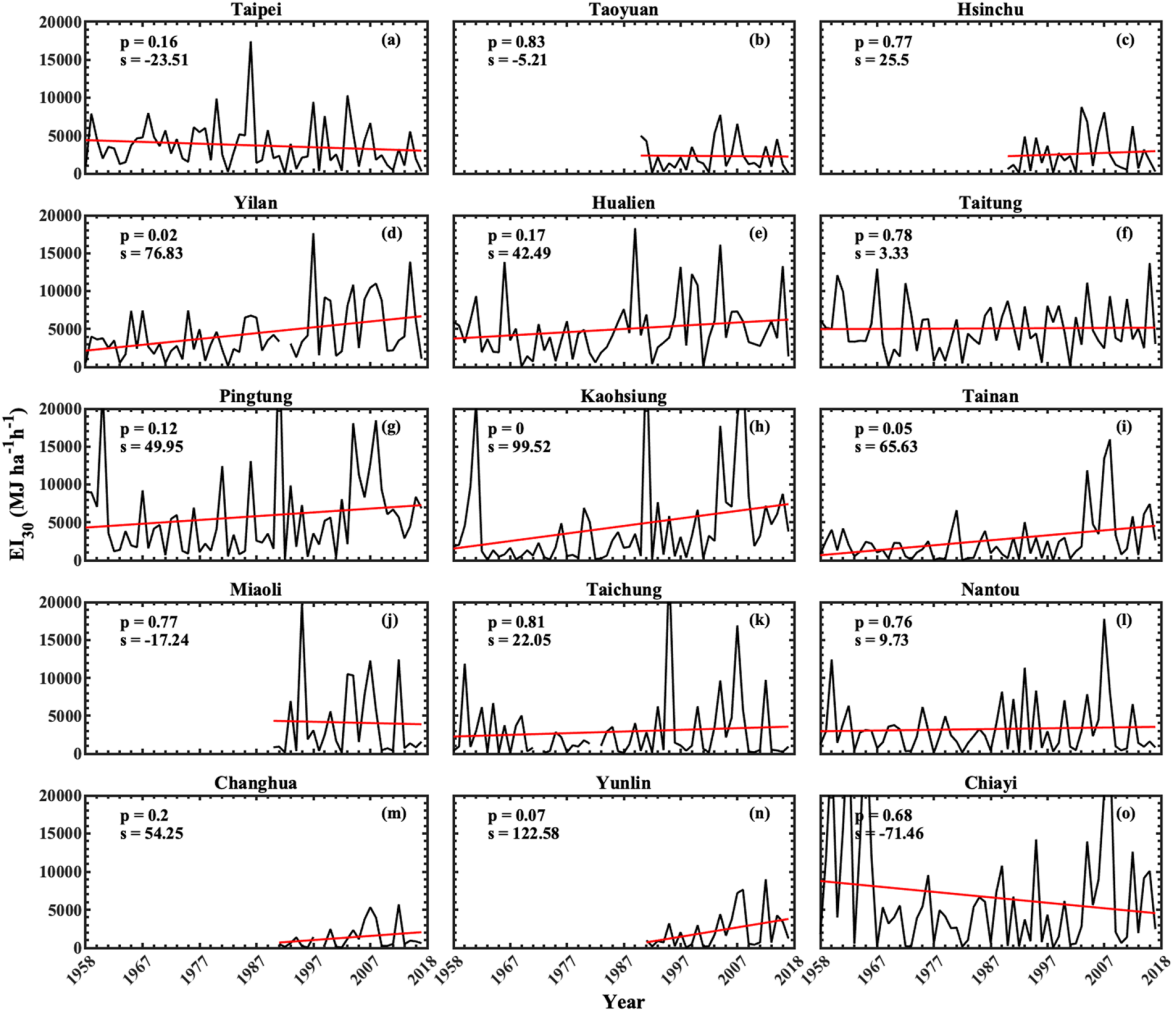
Same as Fig. 4 except for rainfall erosivity (EI_30_).

Because of complex orography of Taiwan^43^, we performed trend analysis for Taiwan by considering different regions/counties rather than complete Island. Regions of this Island are classified into north (which include Taipei, Hsinchu, Taoyuan counties), central (Miaoli, Taichung, Changhua, Nantou, Yunlin, Chiayi), south (Tainan, Kaohsiung, Pingtung), and east (Yilan, Hualien, Taitung), and these regions are depicted with different color in Supplementary Fig. 1b. Trends in typhoons-induced annual mean precipitation and the corresponding erosivity for 15 counties of Taiwan are illustrated in Figs 4 and 5, respectively. The linear trends for all 15 counties are performed by least square regression analysis. A trend is considered as significant if ‘p’ value is less than 0.1 (90% confidence level).

Over north Taiwan, for the annual mean precipitations of typhoons-induced rainfall, except for Taipei county, which shows statistically significant decreasing trend (Fig. 4a), the other two counties (Taoyuan and Hsinchu; Fig. 4b,c) show slightly raising trends. For eastern Taiwan, Yilan and Hualien counties show raising trend (Fig. 4d,e) and the other county (Taitung) shows decreasing trend (Fig. 4f). On the other hand, all the three counties of south Taiwan show raising trends (Fig. 4g–i), with statistically significant raising trend only at Kaohsiung. Over Central Taiwan, except for Chiayi and Miaoli, which show decreasing trend (Fig. 4j,o), the other four counties (Miaoli, Taichung, Nantou, Changhua, and Yunlin (Fig. 4k–n) show raising trends, with statistically significant only at Yunlin.

Figure 5 depicts the time series of typhoons-induced rainfall erosivity for 15 counties of Taiwan. For north Taiwan, Taipei and Taoyuan show decreasing trends (Fig. 5a,b) and Hsinchu shows raising trend (Fig. 5c). However, the trends at these three counties are statistically insignificant. Increasing trends can be noticed for the three counties of eastern Taiwan (Fig. 5d–f), nevertheless, the trend is statistically significant only at Yilan county. Raising trends are also found for the three counties of south Taiwan (Fig. 5g–i) with statistically insignificant at Pingtung and significant at Kaohsiung and Tainan. Among six counties of central Taiwan, statistically significant raising trend at Yunlin (Fig. 5n), statistically insignificant decreasing trends at Miaoli (Fig. 5j) and Chiayi (Fig. 5o), and increasing trends at Taichung (Fig. 5k), Nantou (Fig. 5l) and Changhua (Fig. 5m) can be seen.

## Discussion

In computation of rainfall erosivity, most of the previous studies used either long-term rainfall data of few rain gauge stations^47,52,62^ or short-term data with dense rain gauges^26,27,29^ by using elsewhere KE-I relations rather than adopting region specific relations. Conversely, some researchers used modified Fournier index^30^ in estimation of erosivity, if there were no records of high temporal resolution precipitation data, and only few studies adopted the region specific KE-I relation in the reckoning of erosivity^33^. Nonetheless, globally, least attention was paid for the tropical cyclones-induced rainfall erosivity. Taiwan being most tropical cyclones affecting country, it is paramount to investigate the R-factor for typhoons rainfall.

In this study we used long-term raindrop size distribution information of typhoons to estimate the KE-I relations, and we establish that power law is more appropriate in estimating the Taiwan typhoons R-factor. The estimated KE-I relation is adopted in computing the typhoons R-factor with 60 years (1958–2017) of hourly rainfall data over Taiwan. The annual mean typhoons precipitation for Taiwan is noticed as 586.90 (SD 155.44) mm yr^−1^ with annual mean typhoons rainfall erosivity of 4905.83 (SD 1882.69) MJ mm ha^−1^ h^−1^ yr^−1^, which is much higher than the global mean^31^. Higher R-factor values are found over eastern and southern part of Taiwan, and higher R-factor density values are perceived for southern Taiwan. The typhoons invading region of Taiwan is surrounded by two major typhoons tracks in east and south direction (see Fig. 1 of Tu *et al*.^44^), and the interaction of typhoons that pass through these two major tracks interact with the CMR of the island resulting in an enormous rainfall amounts over eastern and southern region of Taiwan.

The county wise trend analysis performed for Taiwan showed increasing trends in rainfall and erosivity of typhoon events for eastern and southern Taiwan. Over northwest Pacific, during 1977–2013, Mei and Xie^54^ performed the trend analysis for frequency of four types of typhoon clusters (cluster 1–4). Among the four clusters, they demonstrated an increase in high intense (category 4 and 5) typhoons of cluster-1 and cluster-2, which are covered respectively, by eastern and southern Taiwan (Supplementary Fig. 8 of Mei and Xie^54^). An increasing trends in rainfall and erosivity over the eastern and southern region of this island is due to an increase in high intense, long-lasting typhoons and their shift to northward^44,54,58^.

In analyzing the trends for Taiwan, we consider all the typhoons that occurred throughout the year (Jan-Dec) during 1958–2017, and these typhoons should met an erosive event criteria as mentioned in methods sections. Also, we selected the rain gauge stations that are available for greater than or equal to 20 years of observation (≥20 years) during typhoon-induced rainfall periods of 1958–2017. It should be noted that the typhoons whose rainfall amounts are not meeting the erosive event criteria are missing in the trend analysis. Moreover, because of complex topography of Taiwan, a given typhoon need not to produce the rainfall for the complete Island. Further, for a given typhoon-induced rainfall, all the rain gauges distributed over Taiwan may not get a rainfall event that satisfy the erosive event criteria, hence, some rain gauge stations record a rainfall event and some may not. Although there were reports on trends for typhoon-induced rainfall at different regions of Taiwan^59^, because of the above mentioned reasons, rainfall erosive events are not the representative of total rainfall of typhoons and their characteristics need not to be similar to that of the total rainfall amounts. Our findings can contribute to better assessment of soil erosion modeling, agricultural, effective-land use and flood risk assessment for Taiwan.

## Methods

### Study area

Taiwan is a subtropical island in the Western North Pacific (~36197 km^2^) with geological composition of sedimentary and metamorphic rocks, and deep topographic CMR of average height 2 km. Taiwan is influenced by highest annual frequency of tropical cyclones which are contributing to 47.5% of the total annual rainfall^63^. The geographical location of Taiwan with its topography, 711 rain gauge stations, and the Joss-Waldvogel disdrometer (JWD)^64^ is shown in Supplementary Fig. 1.

### Typhoons tracks data

The typhoon track data is based on the World Meteorological Organization’s Joint Typhoon Warning Center (JTWC) (http://www.usno.navy.mil/NOOC/nmfc-ph/RSS/jtwc/best_tracks/). The JTWC provides 6-hourly records of typhoon track information.

### Disdrometer data selection criteria for typhoons rainfall

During 2002–2017, the raindrop size distribution measurements of disdrometer (1-min sample data) are treated as typhoon-induced rainfall when typhoon center (supplementary Fig. 2) is within a radius of 500 km from the disdrometer site^65^ and they are within the CWB typhoon warning periods.

### Definition of erosive rainfall event

A rainfall episode with minimum duration of 30-min and rainfall depth of greater than or nearly equal to 12.5 mm and a 6-hours of continuous non-rainfall gap between two consecutive rainfall episodes is considered as erosive rainfall event^13^. Here we use erosive rainfall event criteria with rainfall depth of greater than or nearly equal to 10 mm. With this rainfall classification criteria, a total number of 75 rainfall events from 66 typhoons are identified from the disdrometer measurements for the period 2002–2017.

### Validation of Disdrometer data

The disdrometer rainfall amounts of selected typhoon rainfall events are validated by comparing with collocated rain gauge. The scatter plot of event rainfall depths between disdrometer and collocated rain gauge is depicted in Supplementary Fig. 3. A good correlation is found between the rain gauge and disdrometer measurements.

### Computation of rainfall intensity (I) and kinetic energy (KE_mm_ and KE_time_)

The rainfall intensity (I, mm h^−1^), kinetic energy expenditure (KE_time_, J m^−2^ h^−1^), and kinetic energy content (KE_mm_, J m^−2^ mm^−1^) for 75 typhoon rainfall events are computed from the RSD information of JWD.

The rainfall kinetic energy (KE) is half the product of raindrop mass and the square of its velocity, and can be expressed in two forms as time specific kinetic energy (KE_time_, in J m^−2^ h^−1^) and volume specific kinetic energy (KE_mm_, J m^−2^ mm^−1^). KE_time_ is kinetic energy per unit area per hour and KE_mm_ is the kinetic energy per unit area per unit depth^18–21^.

The rainfall intensity and kinetic energy are computed by using below equations.

Rainfall intensity (I, mm h^−1^),

1 $${\rm{I}}=(\frac{{\rm{\pi }}}{6})(\frac{3.6}{{10}^{3}})(\frac{1}{{\rm{A}}\,{\rm{T}}})\mathop{\sum }\limits_{{\rm{i}}=1}^{20}\,{{\rm{n}}}_{{\rm{i}}}{{\rm{D}}}_{{\rm{i}}}^{3}$$

Kinetic energy expenditure (KE_time_, J m^−2^ h^−1^),

2 $${{\rm{KE}}}_{{\rm{time}}}=(\frac{{\rm{\pi }}}{12})(\frac{1}{{10}^{6}})(\frac{3600}{{\rm{T}}})(\frac{1}{{\rm{A}}})\mathop{\sum }\limits_{{\rm{i}}=1}^{20}\,{{\rm{n}}}_{{\rm{i}}}{{\rm{D}}}_{{\rm{i}}}\,{\rm{V}}{({{\rm{D}}}_{{\rm{i}}})}^{2}$$

Kinetic energy content (KE_mm_, J m^−2^ mm^−1^),

3 $${{\rm{KE}}}_{{\rm{mm}}}=\frac{{{\rm{KE}}}_{{\rm{time}}}}{{\rm{I}}}$$

where A = 0.005 m^2^ is the sampling area of the sensor, T = 60 s is the sampling time, n_i_ the number of drops of diameter D_i_, V(D_i_) is the fall velocity of drops with diameter (D_i_)^66^.

In this study, data points with rainfall intensities less than 0.1 mm h^−1^ are discarded^67^. The KE_time_-I and KE_mm_-I empirical relations are derived for linear, exponential, logarithmic, and power laws by using non-linear regression analysis.

### Rain gauge data selection criteria for typhoons rainfall

For the period of 1958–2017, hourly rain gauges data during central weather Bureau (CWB) typhoon warning periods (http://rdc28.cwb.gov.tw/TDB/ntdb/pageControl/typhoon) of Taiwan are considered as typhoons attributed rainfall.

### Rain gauge stations selection criteria

In this study, we selected the rain gauge stations with minimum record of 20 years for the estimation of R-factor, with this threshold, among the dense network of 711 rain gauge stations distributed over Taiwan, a total number of 288 stations are qualified for typhoons rainfall record periods of 20 years or higher during 1958–2017. At these 288 rain gauge station, during 1958–2017, a typhoon-induced rainfall amount is considered for further analysis (estimation of R-factor and R-factor density) if it satisfy the erosive rainfall event criteria as mentioned above.

### Calculation of R-factor (EI_30_)

The average annual typhoon rainfall erosivity (R) is computed as:

4 $$R=\frac{1}{{\rm{n}}}\mathop{\sum }\limits_{{\rm{j}}=1}^{{\rm{n}}}\,\mathop{\sum }\limits_{{\rm{k}}=1}^{{{\rm{m}}}_{{\rm{j}}}}\,{({{\rm{EI}}}_{30})}_{{\rm{k}}}$$

where R-factor is the average annual rainfall erosivity (MJ mm ha^−1^ h^−1^ yr^−1^), n is the number of years of record, m_j_ is the number of erosive events of a given year j, and EI_30_ is the typhoon rainfall erosivity index of a single event k.

The single event erosivity EI_30_ (MJ mm ha^−1^ h^−1^) is defined as:

5 $${\rm{EI}}={{\rm{EI}}}_{30}={{\rm{eI}}}_{30}$$

where I_30_ is the maximum 30-min rainfall intensity (I, mm h^−1^) and is calculated by adopting the method of Yin *et al*.^68^. Where e is the rainfall kinetic energy per unit depth of rain and is obtained by using power law of KE_time_-I relation:

$${{\rm{KE}}}_{{\rm{time}}}={{\rm{aI}}}^{{\rm{b}}}$$

where ‘a’ and ‘b’ are empirical coefficients

6 $${\rm{e}}=\frac{{{\rm{KE}}}_{{\rm{time}}}}{{\rm{I}}}=\frac{{{\rm{aI}}}^{{\rm{b}}}}{{\rm{I}}}={{\rm{aI}}}^{{\rm{b}}-1}$$

Annual R-factor values are used to drive rainfall erosivity maps by using ordinary kriging.

The annual mean precipitation value at each rain gauge station is computed by using the expression as mentioned below.

7 $${\rm{Annual}}\,{\rm{mean}}\,{\rm{precipitation}}\,{\rm{at}}\,{\rm{each}}\,{\rm{rain}}\,{\rm{gauge}}\,{\rm{station}}=\frac{{\sum }_{j=1}^{m}{\sum }_{i=1}^{n}\,{P}_{i,j}}{m}$$

where ‘n’ represents the number of events in a given year for a given station, and ‘m’ represents the number of years that have typhoon-induced rainfall events for a given station, and “P_i,j_” is typhoon-induced rainfall accumulation of n^th^ event in m^th^ year.

The annual mean EI_30_ value at each rain gauge station is computed by using the expression as mentioned below.

8 $${\rm{Annual}}\,{\rm{mean}}\,{{\rm{EI}}}_{30}\,{\rm{at}}\,{\rm{each}}\,{\rm{rain}}\,{\rm{gauge}}\,{\rm{station}}=\frac{{\sum }_{j=1}^{m}\,{\sum }_{i=1}^{n}\,{X}_{i,j}}{m}$$

where ‘n’ represents the number of events in a given year for a given station, and ‘m’ represents the number of years that have typhoon-induced rainfall events for a given station, and “X_i,j_” is EI_30_ of a typhoon-induced rainfall of n^th^ event in m^th^ year.

Kriging interpolation is applied to annual mean precipitation and annual EI_30_ values available at Island wide distributed 288 rain gauge stations (whose record period ≥ 20 years during 1958–2017) to plot the spatial distribution (Island-wide gridded data) map for annual mean precipitation and annual EI_30_.

## Supplementary information

05_Supplementary material_R2
