# Supplementary material for: An assessment of tropical cyclones rainfall erosivity for Taiwan
Source: Sci Rep. 2019 Nov 1;9:15862. doi: 10.1038/s41598-019-52028-5 (PMC6825239; doi:10.1038/s41598-019-52028-5)
Supplement: Supplementary file 1 — 05_Supplementary material_R2 [file 41598_2019_52028_MOESM1_ESM.docx]

**An assessment of tropical cyclones rainfall erosivity for Taiwan**

Jayalakshmi Janapati^1,^ ^†^, Balaji Kumar Seela^1, 2, †^, Pay-Liam Lin^1, 3*^, Pao K. Wang^4,5^ ,& Utpal Kumar^6^

^1^Institute of Atmospheric Physics, Department of Atmospheric Sciences, National Central University, Jhong-Li Region, Tao-Yuan City, Taiwan.

^2^Taiwan International Graduate Program (TIGP), Earth System Science Program, Research Center for Environmental Changes, Academia Sinica, Taipei, Taiwan

^3^Earthquake-Disaster & Risk Evaluation and Management Center, National Central University, Jhong-Li Region, Tao-yuan City, Taiwan.

^4^Department of Atmospheric and Oceanic Sciences, University of Wisconsin-Madison, Madison, Wisconsin, USA,

^5^Research Center for Environmental Changes, Academia Sinica, Taipei, Taiwan.

^6^Taiwan International Graduate Program (TIGP), Earth System Science Program, Institute of Earth Sciences, Academia Sinica, Taipei, National Central University, Taiwan

† Equal contribution of authors.

***Corresponding Author and address:**

Prof. Pay-Liam Lin,

Department of Atmospheric Science,

College of Earth Sciences, National Central University, Jhongli City, Taiwan.

Phone: 03-422-3294 03-422-7151 ext. 65509

E-mail: [tliam@pblap.atm.ncu.edu.tw](mailto:tliam@pblap.atm.ncu.edu.tw)

**Supplementary Figures:**


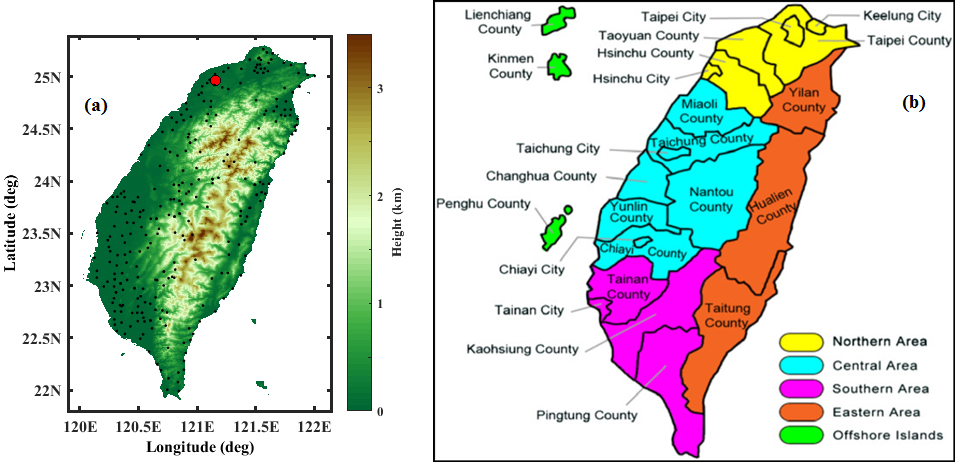


**Supplementary Figure 1.** (a) Geographical locations of Taiwan with the location of Joss-Waldvogel disdrometer represented with red color filled circle, and the rain gauge stations denoted with black dots. (b) Map showing the different regions (north, south, east and central) of Taiwan with county names.


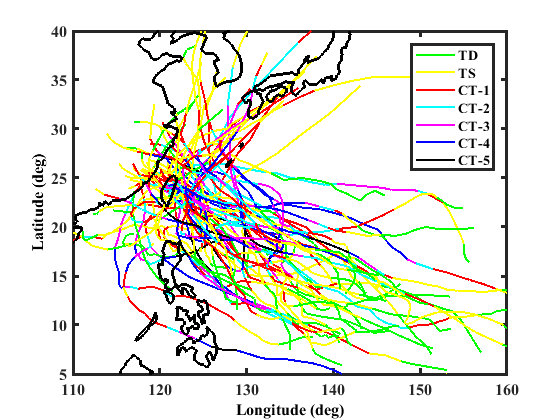


**Supplementary Figure 2.** Track of typhoons during 2002-2107, that were recorded by Joss-Waldvogel disdrometer.


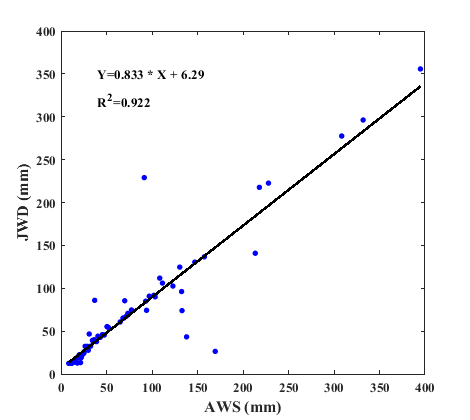


**Supplementary Figure 3.** Validation of disdrometer measured typhoon events rainfall with collocated rain gauge rainfall.


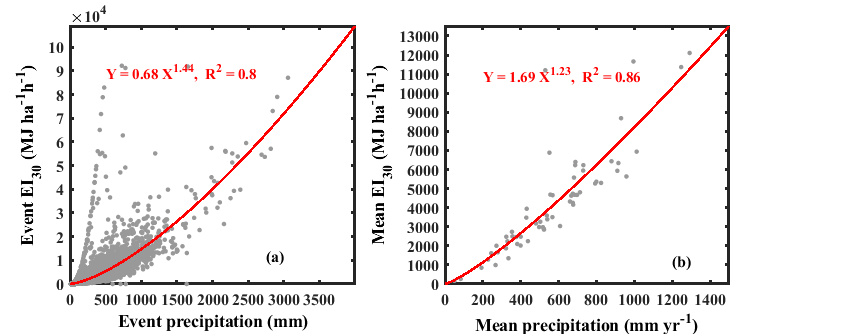


**Supplementary Figure 4.** Relation between (a) events precipitation and EI_30_ (b) annual precipitation and EI_30_. The date points in the above figure panels corresponds to typhoons-induced rainfall over Taiwan during 1958-2017.
